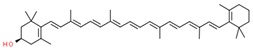# Antioxidant Therapies for Neuroprotection—A Review

**DOI:** 10.3390/jcm8101659

**Published:** 2019-10-11

**Authors:** Raluca Ioana Teleanu, Cristina Chircov, Alexandru Mihai Grumezescu, Adrian Volceanov, Daniel Mihai Teleanu

**Affiliations:** 1 “Victor Gomoiu” Clinical Children’s Hospital, “Carol Davila” University of Medicine and Pharmacy, 050474 Bucharest, Romania; raluca.teleanu@umfcd.ro; 2 Department of Science and Engineering of Oxide Materials and Nanomaterials, Faculty of Applied Chemistry and Materials Science, Politehnica University of Bucharest, 011061 Bucharest, Romania; cristina.chircov@yahoo.com (C.C.); grumezescu@yahoo.com (A.M.G.); 3 Emergency University Hospital, “Carol Davila” University of Medicine and Pharmacy, 050474 Bucharest, Romania; daniel.teleanu@umfcd.ro

**Keywords:** antioxidant therapy, reactive nitrogen species, reactive oxygen species, neuroprotection

## Abstract

Although moderate concentrations of reactive oxygen species (ROS) and reactive nitrogen species (RNS) are crucial for various physiological processes within the human body, their overproduction leads to oxidative stress, defined as the imbalance between the production and accumulation of ROS and the ability of the body to neutralize and eliminate them. In the brain, oxidative stress exhibits significant effects, due to its increased metabolical activity and limited cellular regeneration. Thus, oxidative stress is a major factor in the progressive loss of neurons structures and functions, leading to the development of severe neurodegenerative disorders. In this context, recent years have witnessed tremendous advancements in the field of antioxidant therapies, with a special emphasis for neuroprotection. The aim of this paper is to provide an overview of the oxidative stress and antioxidant defense mechanisms and to present the most recent studies on antioxidant therapies for neuroprotection.

## 1. Introduction

Reactive oxygen species (ROS) and reactive nitrogen species (RNS) are chemically reactive molecules that include radical and non-radical oxygen species normally produced through the partial reduction of oxygen, e.g., hydrogen peroxide, hydroxyl radical, superoxide radical anion, nitric oxide, and peroxynitrite [1,2]. The main source of these species is the mitochondrial oxidative phosphorylation through an electron leakage from the mitochondrial electron transport chain that results in the formation of free oxygen radicals. Additionally, such molecules are also produced through the action of certain enzymes, including NADH, NADPH oxidase, xanthine oxidase, cyclooxygenases, and lipoxygenases. Subsequently, reactive species can lead to the production of reactive aldehydes, malondialdehyde, and 4-hydroxynonenal [1,3,4].

While moderate concentrations of ROS play pivotal roles in several physiological processes, including cell cycle regulation, inflammation, phagocytosis, enzyme and receptor activation, stressor responses, gene expression, and signal transduction, their overproduction can lead to serious pathological damages [3,4,5,6,7,8]. Specifically, high concentrations of ROS lead to oxidative stress, which is defined as a disparity of the balance between the production and accumulation of ROS within cells and tissues, and the ability of the biological system to neutralize them and detoxify these by-products [3,6,7,9]. The factors that could influence the increase of ROS production are represented by environmental stressors, such as UV, ionizing radiations, heavy metals, and pollutants, or xenobiotics, such as antiblastic drugs [6,10]. DNA/RNA, proteins, and lipids represent the cellular molecules mainly targeted by oxidative stress, and their modification can potentially lead to mutagenesis. Furthermore, the cellular structure and function are damaged, leading to preneoplastic and neoplastic transformations or cell death through necrosis and apoptosis [3,5,11]. Therefore, oxidative stress has been correlated with the development of numerous diseases, including cancer, arthritis, diabetes, autoimmune disorders, muscle dysfunctions, and allergies [4,5,7,12]. Additionally, as age-associated functional losses are connected with the accumulation ROS/RNS-induced damages, oxidative stress is involved in the development of various age-related conditions, including neurodegenerative disorders, cardiovascular diseases, obstructive pulmonary disease, chronic kidney disease, sarcopenia, frailty, cancer, diabetes mellitus, autoimmune rheumatic diseases, and infectious diseases [13,14,15,16]. However, the effects of ROS/RNS are concentration-dependent. Specifically, such as in the case of nitric oxide, the dichotomic action of ROS/RNS can be observed in cancer cells, where they can either act as secondary messengers in the intracellular signaling cascades, inducing and/or maintaining the oncogenic phenotype, or they can induce the senescence and apoptosis of the cells, acting as anti-tumorigenic molecules [17,18,19,20,21]. Similarly, nitric oxide can function both as a pro-inflammatory and an anti-inflammatory molecule in the cell regulation processes [22,23].

The effects of oxidative stress on the brain are especially significant as it is a highly metabolically active organ with limited cellular regeneration capacity [4]. Although it comprises 2% of the body weight, it consumes 20% of the body oxygen owing to its high rate of metabolism, which subsequently leads to a higher oxygen availability. These high levels of oxygen consumption are required for the ATP generation by providing an efficient electron acceptor for several physiological processes, including action potentials, neurotransmission, synaptic machinery for transmitters exocytosis and energetic pump maintenance, and enzymatic reactions. The vulnerability of the brain to oxidative stress is caused by endogenous factors, such as modest antioxidant defenses, the dependence on excitotoxic and auto-oxidizable neurotransmitters, glymphatic waste disposal, peroxidation susceptibility of polyunsaturated fatty acids, calcium load, redox-active metal burden, and limited regenerative capacity [24,25]. As oxidative stress triggers several molecular pathways leading to the progressive loss of neuronal structures and functions, a process termed neurodegeneration, it is considered a major factor in the development of neurodegenerative disorders, including multiple sclerosis, amyotrophic lateral sclerosis, Alzheimer’s disease, Parkinson’s disease, and Huntington’s disease [7,24,26,27,28].

Considering the essential role of oxidative stress in neuronal disorders, there has been a growing interest for the development of antioxidant therapeutics that could potentially mimic the physiological processes of the natural antioxidant defensive system, which is mainly based on enzymatic components, including superoxide dismutase, catalase, and glutathione peroxidase [6,13,24,29,30]. Specifically, there has been a considerable scientific focus on the application of antioxidants for neuroprotection, which is a treatment alternative for central nervous system disorders that targets oxidative stress and excitotoxicity [31]. Antioxidants are natural or synthetic chemical substances capable of averting or removing oxidative stress-related diseases by counteracting the negative effects of ROS/RNS at low concentrations [25,32,33]. The beneficial effects of antioxidants are related to their anti-inflammatory, anti-bacterial, anti-cancer, cardioprotective, and neuroprotective properties, and there is evidence supporting their potential therapeutic effects on diabetes, arthritis, osteoporosis, and cataracts [6,34,35]. However, the number of drugs with antioxidant properties approved for human use in central nervous system disorders is still limited, which requires more intensive work in the area of antioxidant drugs development [24,36,37]. Additionally, the dichotomic properties of ROS/RNS are challenging for the selection of suitable antioxidant agents. In this context, the aim of this paper is to provide an overview of the oxidative stress mechanisms and the currently available antioxidant therapies for neuroprotection.

## 2. Oxidative Stress Mechanisms

As initially theorized by Selye in 1956 and later confirmed by Holmes and Rahe in 1967, stress is defined as an organism’s response to a threatening stimulus in order to regain homeostasis [38]. Later, in 1985, the concept of oxidative stress was formulated, meaning a “a disturbance in the prooxidant-antioxidant balance in favor of the former” [39]. The biological antioxidants causing oxidative stress include the by-products of endogenous and exogenous processes that involve oxygen and nitrogen [28,40]. Although molecular oxygen is kinetically unreactive, it is a thermodynamically strong oxidant due to its electron structure that comprises two unpaired electrons in its basic triplet state in the two antibonding π* orbitals [28,41].

The sources for the generation of ROS can be mainly divided into endogenous, which include biological processes that release ROS as by-products, such as the mitochondrial electron transport chain, the endoplasmic reticulum, microsomes and peroxisomes, membrane-bound NADPH oxidase (NOX) family enzymes, and nitric oxide synthases; and exogenous, represented by the cellular processes as responses to bacterial invasions, cytokines, and xenobiotics, due to oxidative burst activity in macrophages [10,28,41,42].

The majority of free radicals in cells are generated by the mitochondrial oxidative phosphorylation, through the electron leak from the electron transport chain [43,44]. Mitochondria are unique double-membrane bound organelles present within eukaryotic cells acting as energy centers of the cells [45]. Specifically, the principal role of mitochondria is to produce metabolic energy in the form of ATP through the oxidative phosphorylation, which occurs after the oxidation of the reduced co-enzymes involved in the respiratory chain. The process of aerobic oxidation is based on the citric acid cycle interconnection, which represents the final metabolic pathway for all major nutrients oxidation [46,47]. In this context, the transmembrane protein complexes and the electron transport carriers, i.e., cytochrome c and ubiquinone, which comprise the electron transport chain and exist in the cristae of the mitochondria, must be specifically assembled into a supercomplex to produce ATP together with the F1F0-ATP synthase enzyme [48]. In most cases, small molecule electron carriers such as NADH, NADPH, reduced coenzyme, and reduced glutathione do not react with O_2_, but rather regenerate it [46]. Initially, the superoxide radical is formed at complexes I and III of the electron transport chain through the single electron reduction of O_2_ via the H^+^ pumps of the respiratory chain, on the redox-active prosthetic groups of electron-binding proteins, such as reduced coenzyme [10,41,46]. Since this reduction is thermodynamically more advantageous, there are multiple electron donors within the mitochondria to allow for this reaction [46]. Moreover, it is estimated that 1% of the total O_2_ consumption of the mitochondria is used for the generation of the superoxide radical [44,49]. The leak of such electrons from redox centers or enzyme subunits or complexes results in the formation of ROS in the mitochondrial matrix or intermembrane space. The superoxide radical is rapidly transformed by the superoxide dismutase enzyme into the non-radical ROS H_2_O_2_, which permeates the membrane and diffuses between cellular compartments [10,42,43,46,47]. Subsequently, the hydroxyl radical is produced through a respiratory burst via the Fenton reaction or the Haber-Weiss reaction from H_2_O_2_ and metal species, such as iron or copper [44,50,51]. Similarly, ROS can be generated by the myeloperoxidase–halide–H_2_O_2_ system. In the presence of the chlorine ion, H_2_O_2_ is converted to hypochlorous by the action of the myeloperoxidase enzyme, which is found in the neutrophil cytoplasmic granules [51]. The availability of substrates and final electron acceptors of the electron transport, the rate of substrates production in the citric acid cycle and ATP consumption, and the rate constants of the enzymatic reactions limit the electron transport chain [43]. Furthermore, it has been demonstrated that the production of ROS is strongly dependent on the membrane potential of the mitochondria, as high values result in increased rates of ROS generation [43,52]. An electron leak can also be induced through the inhibition of complex I and III using certain inhibitors, such as rotenone and antimycin A [43].

ROS production within the mitochondria can also occur outside the electron transport chain, through the action of α-ketoglutarate dehydrogenase or pyruvate dehydrogenase complexes. However, a reduced activity of the α-ketoglutarate dehydrogenase complex in the brain has been associated to various neurodegenerative diseases, such as Parkinson’s disease or Alzheimer’s disease. Additionally, hydrogen peroxide formation is stimulated by the catabolism of monoamines by monoamine oxidase (MAO) enzyme family in the homeostasis of neuromodulators and neurotransmitters [43]. The MAO enzyme family can be divided into two isoforms that are implicated in the modulation of the redox-state of neuronal and glial cells, namely MAO-A and MAO-B. Within the brain, MAO-A is mostly found in catecholaminergic neurons, as it oxidizes noradrenaline and serotonin. By contrast, MAO-B is mainly expressed in serotonergic neurons, astrocytes, and glial cells, and it oxidizes beta-phenylethylamine. As they belong to the flavin-containing amine oxidoreductases protein family, their oxidizing activity necessitates the presence of the co-factor flavin adenine dinucleotide, which can bind to the cysteine residue. Using molecular oxygen, MAO enzymes remove an amine group from a molecule to further produce an aldehyde and ammonia, consequently generating H_2_O_2_ as a by-product [53].

Other enzymes that are major endogenous sources of ROS include the NOX, xanthine oxidase, cytochrome P450, and lipoxygenases [10]. The NOX family comprises seven membrane-bound, multi-subunit protein complexes, namely NOX1-5, DUOX1, and DUOX2, which are categorized into three groups, depending on their structural domains, and have a dedicated function to form ROS [10,42,54,55]. The native proteins are inactive and they depend on interacting proteins for their subsequent maturation, stabilization, haem incorporation, and translocation across the membrane, to the site of activity [55]. As their main function is the electron transfer across the plasma membrane, the NOX family catalyzes the production of superoxide anion from O_2_ and, consequently, of ROS [44,55]. NOX1-4 are similar in size and domain structure, NOX5 has a different domain structure but a similar process of superoxide formation, and DUOX1 and DUOX2 contain a peroxidase-homology domain and they utilize the ROS generated by the catalytic core for the production of species with higher oxidative capacities [42]. NOX1-3 interact with p22^phox^ transmembrane protein along with the cytosolic organizer subunits, activator subunits, and the G-protein Rac, NOX4 interacts only with p22^phox^, and the activation of NOX5, DUOX1, and DUOX2 depends on the direct Ca^2+^ binding [55]. When activated, NOX1-3 and NOX5 mainly generate superoxide, while NOX4, DUOX1, and DUOX2 produce H_2_O_2_ directly [10,55]. The factors involved in the production of ROS through the activation of the NOX family include environmental factors, such as hypoxia, mechanical forces, and cytokines and hormones, such as angiotensin II, aldosterone, endothelin-1, platelet-derived growth factor, transforming growth factor β, and tumor necrosis factor α [42,55]. As each of the isoforms have been related to different diseases, i.e., NOX1, to diabetic atherosclerosis and retinopathy, NOX2, to neurodegeneration, NOX4, to stroke, diabetic nephropathy, and neuropathic pain, and NOX5, to diabetic nephropathy, hypertension, and coronary artery disease, isoform-specific targeting could represent a treatment solution. Thus, the development of pharmacological formulations containing isoform-selective NOX inhibitors could lead to improvements in the therapy of various diseases [56].

Furthermore, nitric oxide is an oxygen-derived free radical with an unpaired electron centered at the nitrogen atom [41,53]. It is involved in various vital processes, including vascular and airway tone, immune defense, insulin secretion, peristalsis, and angiogenesis, by targeting haem proteins and reacting with thiols and oxygen ions [28,41,53]. Additionally, it plays fundamental roles within the central nervous system, including neural development, cerebral blood flow regulation, and memory [28]. Nitric oxide is produced in the mitochondria through the oxidation of L-arginine to L-citrulline, with the consumption of molecular oxygen and NADPH in the process [28,41,44,51,53], and it inhibits complex IV of the electron transport chain [57]. The reaction is catalyzed by three main nitric oxide synthase (NOS) isoforms, namely the Ca^2+^/calmodulin-dependent neuronal NOS which is expressed in astrocytes, microglia, and macrophages, the endothelial NOS, expressed mainly by endothelial cells within the vascular endothelium, and the Ca^2+^-independent inducible NOS, expressed in immune cells [28,41,44]. The activity of the NOS isoforms is highly dependent on NADPH, tetrahydrobiopterin, and molecular oxygen as co-factors [53]. While nitric oxide is an RNS, it can directly interact with superoxide to form peroxynitrite, which is a highly reactive compound [44,51,53]. Peroxynitrite is involved in a variety of damaging effects, including lipid peroxidation, enzyme inactivation, and mitochondrial respiration inhibition, as it targets proteins and irreversibly affects their structure and function, a process known as nitrosative stress [41,44,51]. Peroxynitrite can also nitrate and oxidize adenine, guanine, and xanthine nucleosides. Moreover, low concentrations have been associated to apoptotic cell death, while high concentrations have been related to necrosis [44].

The hydroxyl radical and peroxynitrite are the major endogenous oxidants that cause damage to the nucleic acids, including nucleotide bases modifications, apurinic and apyrimidinic sites training, and single or double strand breaking. Furthermore, guanine is the most susceptible nucleotide base due to its lower reduction potential that results in the interactions with the imidazole ring at positions C4, C5, and C8 [58]. Nevertheless, ROS and RNS can lead to excessive lipid peroxidation, which is a highly cell damaging process. Through the formation of lipid hydroperoxides and aldehydes, e.g., malondialdehyde, 4-hydroxynonenal and isoprostanes, cellular toxicity increases, as the normal structure and function of the lipid bilayers are disrupted, which consequently results in membrane permeability, transportation, and fluidity alterations [58]. The phospholipids abundantly found in the brain are considerably vulnerable to peroxidation caused by ROS and RNS, which has been associated to the development of many central nervous system disorders [59,60]. Moreover, as proteins represent the major non-water components of biological systems, they are a significant target of oxidative stress. The main mechanisms that affect amino acids, peptides, and proteins are hydrogen abstraction, electron transfer, addition, fragmentation and rearrangement, dimerization, disproportionation, and substitution reactions [61]. The effects of these processes have been associated to impaired protein folding, side-chain oxidation, and backbone fragmentation, which subsequently leads to function loss and biochemical processes stopping [58]. Additionally, the neurotoxic aggregation of specific proteins in the brain have been associated to the development of neurodegenerative diseases, specifically misfolded tau and amyloid β proteins in Alzheimer’s disease, α-synuclein proteins in Parkinson’s disease, and mutant Huntington proteins in Huntington’s diseases [60].

## 3. Antioxidant Defense Mechanism

As an increase of the ROS/RNS levels has been related to several diseases in the human body, such as cancer, inflammatory diseases, or neurological disorders [62,63], the activity of the natural defense mechanisms comprising antioxidant and detoxifying enzymes is critical [64]. Antioxidants are chemical molecules responsible for protecting the body against oxidative stress by preventing or reducing the oxidation process of macromolecules [62,64,65,66]. Specifically, the toxicity of ROS/RNS is counteracted by the action of a diverse group of bioactive molecules that inhibit oxidation reactions by being oxidized themselves [63,64,67,68].

Antioxidants can be classified based on several factors, namely their source, i.e., natural and synthetic antioxidants, their solubility, i.e., oil-soluble and water-soluble antioxidants, and their mechanism of action, i.e., primary or radical scavengers, secondary or peroxide decomposers, and metal deactivators [69]. However, antioxidants are generally categorized into endogenous compounds and exogenous, either natural or synthetic [35]. The endogenous antioxidants are the naturally occurring molecules that counteract the oxidative stress through several physiological processes [30]. Endogenous antioxidants can be further classified into enzymatic, i.e. superoxide dismutase, glutathione peroxidase, catalase, and glutathione reductase, non-enzymatic, i.e., glutathione, thioredoxin, ferritin, transferrin, ceruloplasmin, albumin, and metallothionein, enzyme co-factors, i.e., coenzyme Q and alpha-lipoic acid, and metabolites, i.e., bilirubin, melatonin, and uric acid [28,30,32,33,50,64,66,67]. Exogenous antioxidants are dietary compounds, comprising natural antioxidants, such as vitamins A, E, and C, flavonoids, phenolic acids, and carotenoids, and synthetic antioxidants, such as butylated hydroxytoluene, octyl gallate, butylated hydroxyanisole, propyl gallate, tert-butylhydroquinone, and ethylenediaminetetraacetic acid [28,33,70].

Within the body, antioxidants aim to prevent the reactions between free radicals and biological compounds, interrupt the radical oxidation reaction, or inactivate the free radical products by repairing the structural damage [32,71]. In this context, the main antioxidative mechanisms involve transition metal ions chelation, ROS and RNS scavenging and quenching, free radical chain reaction breaking for preventing the production of toxic metabolites and mediators of inflammation, molecular repairing, and antioxidant defense system initiation and enhancing by regenerating other antioxidants and regulating enzyme activities [50,63,64,67]. Hence, antioxidants can be subsequently classified into primary or chain-breaking antioxidants, which directly react with free radicals and transform them into stable, non-radical compounds, and secondary, or preventive antioxidants, which are responsible for metal ion chelation and singlet-oxygen quenching and scavenging, or for the regeneration of primary antioxidants and the restoration of their antioxidant activity [70].

Superoxide dismutase is a metalloprotein which catalyzes the dismutation of superoxide into hydrogen peroxide by transferring an electron from one superoxide molecule to another [72,73,74,75]. Specifically, as the donor molecule becomes dioxygen, the recipient rapidly combines with two hydrogen ions to produce hydrogen peroxide [75]. There are three superoxide dismutase isoforms found in the human body, namely copper/zinc-superoxide dismutase, manganese-superoxide dismutase, and extracellular superoxide dismutase [76], and they are situated in the cytosol and the intermembrane space of mitochondria [73,77]. Hydrogen peroxide is further reduced to water and molecular oxygen through the action of catalase and glutathione peroxidase [71,75]. Catalase is an intracellular antioxidant enzyme, which can be categorized into three classes, including monofunctional catalase or typical catalase, catalase-peroxidase, and pseudocatalase or manganese-catalase [78,79]. Structurally, catalase is a tetrameric haem protein and it is mostly present in peroxisomes [80]. By contrast, glutathione peroxidase is a cytosolic antioxidant enzyme that acts against a wider variety of peroxides, as it plays major roles in preventing lipid peroxidation and maintaining the intracellular homeostasis and redox balance [75,81]. After reducing hydrogen peroxide, the oxidized glutathione further oxidizes thiol groups in proteins [71].

Glutathione is both naturally occurring and fortified by diet antioxidants, comprising glycine, cysteine, and glutamic acid [82]. Within the body, it is generated in the cytoplasm and it is found in most tissues, with high concentrations in the liver [83,84]. Glutathione is the major thiol-disulfide redox buffer of the cell, thus playing crucial roles in detoxification and antioxidant mechanisms of the organism [82,84,85]. The reactions of glutathione are catalyzed glutathione-S-transferase and they involve the conjugation with aliphatic or aromatic epoxides and halides or organic nitrates, which are further reduced to nitrites [83]. Additionally, glutathione is involved in the regeneration of vitamins C and E and carotenoids, the maintenance of the redox state of crucial protein sulfhydryl groups required for DNA repair, and the chelation of transition metals [85,86]. Moreover, studies have shown that patients with Parkinson’s diseases present significantly reduced glutathione levels in the substantia nigra [87].

Nrf2 plays a fundamental role in the redox homeostasis and is considered one of the most important transcription factors for antioxidant responses. Its action is based on regulating the basal expression of various genes involved in the antioxidant defense mechanisms or on inducing their expression in stress conditions [88].

The intense mitochondrial activity within the brain requires high neutralizing capacities of the antioxidant defense mechanism to prevent the accumulation of ROS and RNS and the production of oxidative stress. While astrocytes and the astrocytic Nrf2 pathway play a considerable role in assuring antioxidant support to the surrounding neurons, they are often unable to counteract the oxidative damage of the brain cells [89]. Therefore, there has been great scientific focus on the development of antioxidant therapies for neuroprotection that could possibly prevent the onset of neurodegenerative disorders or provide an effective treatment strategy.

## 4. Antioxidants for Neuroprotection

As previously mentioned, although the endogenous antioxidant defense mechanisms are fundamental in the prevention of oxidative stress-induced damage, they are often overcome by the excessive production of ROS and RNS. In this regard, studies have proved significant beneficial effects of the administration of exogenous antioxidant molecules [90]. These approaches are of substantial importance in the area of neurodegenerative disorders management, as considerable scientific studies have focused on finding substances with antioxidant properties for neuroprotection [91]. The most common antioxidant molecules studied for their neuroprotective effects are summarized in Table 1.

### 4.1. Vitamins A, E, and C

Vitamin A is a class of fat-soluble chemical compounds, known as retinoids, that includes retinol, retinal, and retinoic acid [92,93,94]. Retinol, the most active form, is an important chain-breaking antioxidant, involved in scavenging superoxide and hydroxyl radicals [94]. It is known for its essential role in cellular differentiation, vision [95] and wound healing, and its deficiency in the human body has been associated with multiple pathological conditions, such as inflammatory bowel disease [96]. While vitamin A activity is not mainly involved in the antioxidative mechanisms of the brain, one study demonstrated its neuroprotective effects against amyloid fibrillation-associated cytotoxicity. As it has the ability to interact with the Aβ-42 peptide, vitamin A might represent a potential candidate in the therapy of Alzheimer’s and Parkinson’s diseases by combating systemic amyloidosis [97].

Vitamin E or α-tocopherol is one of the most effective chain-breaking antioxidants and a major ROS scavenger in brain cells by preventing lipid peroxidation [95,98]. Generally, vitamin E is a dietary antioxidant that is further incorporated into lipoproteins and systemically delivered through the activity of α-tocopherol transfer protein [98,99]. As it is able to cross the blood-brain barrier, vitamin E plays fundamental roles within the brain, such as functioning as a survival factor for Purkinje neurons that prevent the onset of ataxia with vitamin E deficiency syndrome, activating neuroglial differentiation, cytoprotecting hippocampal neurons, and regulating the inflammatory pathways of the neuroglia [100]. Studies have shown that Alzheimer’s disease patients present lower levels of vitamin E and, therefore, it has been proposed as a candidate for treatment [101]. Additionally, vitamin E administration has proved to prevent or attenuate ischemia-reperfusion injury [102]. While animal studies performed on Sprague Dawley rats demonstrated the efficiency of vitamin E as a neuroprotective and antioxidant agent in neurodegeneration-induced chronic cerebral hypoperfusion [103], its efficiency is not clear yet. The results of the Prevention of Alzheimer’s Disease by Vitamin E and Selenium clinical trial which involved the administration of vitamin E, selenium, Vitamin E and selenium, or placebo, have demonstrated that vitamin E is not capable of preventing dementia, despite previous in vitro and in vivo studies stating its potential [104]. However, as the antioxidant effects of vitamin E are potentiated by the presence of vitamin C which regenerates the tocopheroxyl radical to its reduced form, the co-administration of these vitamins could improve the neuroprotective effects [98].

Vitamin C, also known as ascorbic acid, is an essential water-soluble antioxidant that performs many important functions in the body [96,105]. It is considered as an ideal antioxidant as it is characterized by a low reduction potential that allows for its reaction with various ROS [106]. Specifically, ascorbate peroxidase uses two molecules of ascorbate to reduce H_2_O_2_ to H_2_O. Additionally, ascorbate is able to react with hydroxyl, peroxyl, and singlet O_2_ radicals and it serves as a co-antioxidant through the regeneration of α-tocopherol from its radical [106,107]. In the body, vitamin C is involved in many physiological processes, such as the formation of collagen as a co-factor [108] and the conversion of cholesterol to bile acids [109]. Moreover, vitamin C is present in the brain in high concentrations, participating in processes such as neuronal differentiation, maturation, and survival, dopamine and norepinephrine biosynthesis, neurotransmission modulation, and neuronal protection against glutamate toxicity [105,110]. However, by contrast to most mammals that produce vitamin C from glucose in the liver, humans cannot synthesize it due to the lack of L-gulonolactone enzyme and are dependent on dietary sources [105,111].

Several studies have shown that vitamin C deficiencies could be related to neurodegenerative disorders, such as Parkinson’s, Alzheimer’s, and Huntington’s diseases and amyotrophic sclerosis [105,110]. Particularly, a clinical study showed that the administration of vitamin C and/or E supplements, resulted in a decrease of the risk of cognitive decline in older persons ≥65 years old [112]. Another study investigated the neuroprotective effects of vitamin C in chronic restraint stress-induced rats. As the results showed significant increases in catalase, superoxide dismutase, and nitric oxide levels with a significant decrease of malondialdehyde concentrations, it can be concluded that oral administration of vitamin C prevents oxidative stress and induces neuroprotection by improving the synaptic activities and cognitive functions. Therefore, it can provide a potential treatment for stress-related cognitive decline [113]. Moreover, a study performed on a Drosophila model with Parkinson’s disease-like phenotypes caused by the knockdown of the Parkinson’s disease-related gene homologue dUCH, which are characterized by locomotive impairments and neurodegeneration, showed that the administration of vitamin C at high doses leads to significant side effects, and long-term treatment could prevent the degeneration of dopaminergic neurons [105]. The effect of vitamin C in lipopolysaccharide-induced cognitive impairment as a model for neuroinflammatory cognitive dysfunction was also investigated. The administration of vitamin C by intracerebroventricular microinjection before lipopolysaccharide exposure has protected the male C57BL/6 mice from cognitive impairments as the antioxidant prevented the activation of microglia and the release of pro-inflammatory cytokines, decreased the malondialdehyde levels, and increased the activity of superoxide dismutase [114]. Moreover, vitamin C has also proved to protect male rat pups against hippocampal neuronal induced by neonatal hypothyroidism [115] and protected cells against methamphetamine toxicity by attenuating ROS production, apoptosis, and autophagy [116].

### 4.2. Phenolic Compounds

Phenolic compounds are the biologically active secondary metabolites most widely distributed in plants where they are derived from the pentose phosphate, shikimate, and phenylpropanoid pathways. By acting at molecular levels, phenolic compounds play fundamental roles in the reproduction, growth, protection, and sensory characteristics of plants [117,118,119]. The chemical structure of these compounds can range from simple phenolic molecules to highly polymerized compounds, comprising a phenol unit, meaning one or more hydroxyl groups attached to an aromatic ring [117,120,121,122,123,124]. Moreover, they can naturally occur as mono- or polysaccharides conjugates or they can be associated with esters and methyl esters [117]. Owing to their structural variability, there are more than 8000 phenolic compounds currently known [117,120]. Phenolic compounds exhibit antioxidative properties owing to their capacity to scavenge free radicals and donate hydrogen atoms, electrons, and chelate metal cations. The antioxidant activity is closely related to their molecular structures, specifically, the number and positions of the hydroxyl groups and the nature of the substitutions on the aromatic rings. It is mostly conferred by the hydrogen atoms of the adjacent hydroxyl groups, the double bonds of the benzene ring, and the double bond of the oxo functional group [125]. They are known for their effects on mediating neuroinflammation and neurodegenerative diseases by targeting the toll-like receptor (TLR), especially the TLR4 pathways. In this manner, phenolic compounds targeting TLR4 could serve as pharmacophores in the development of therapeutic strategies for the treatment of neurological disorders [126,127].

Generally, phenolic compounds are categorized into two main groups, namely flavonoids and non-flavonoids [120]. Flavonoids are abundantly found in fruits, vegetables, cocoa, dark chocolate, and beverages, such as red wine and tea, accounting for almost two-thirds of dietary phenolic compounds [120,128]. They are biosynthesized from acetic acids or phenylalanine derivatives via the shikimic acid pathway. Flavonoids are mostly found in glycosylated or esterified forms, consisting of C_6_-C_3_-C_6_ rings, specifically rings A and B linked through a three-carbon ring C [129]. Depending on the position of the benzopyrone moiety to the aromatic rings, flavonoids are classified into flavonoids (2-phenylbenzopyrans), isoflavonoids (3-benzopyrans), and neoflavonoids (4-benzopyrans) [120,129,130]. Furthermore, they can be categorized into various subgroups depending on the hydroxylation and substitution patterns, the oxidation and saturation degrees, the annularity of ring C, and the connection position of ring B [129,130]. The structure of flavonoids offers them a lipophilic character, which allows for the permeation of the blood-brain barrier. Therefore, the interactions between the cell membranes and flavonoids are fundamentally important as the embedding of flavonoids within the lipid bilayer could lead to modifications amongst the lipid head and/or tails [131,132]. In this manner, flavonoids have been regarded as neuroprotective antioxidants by preventing the formation of free radicals through the modulation of cell signaling pathways involved in cell proliferation and survival, glutathione synthesis, and antioxidative proteins expression [132,133]. Moreover, they exhibit neuroprotective properties through an increase in neuron viability, tissue perfusion, and cerebral blood flow, and have been shown to reduce ischemic-related apoptosis, amyloidogenic effects, and loss of dopaminergic neurons [134]. These effects are believed to result from the ability of flavonoids to form ligands for γ-aminobutyric acid type A (GABA_A_) receptors in the central nervous system [135].

The potential of flavonoids for preventing neurodegeneration has been widely studied. One group reported the investigated action of amurensin and cosmosiin, flavonoids isolated from Trigonella foenum extracts against NaNO_2_-induced neurodegeneration in mice brains, proving the potential of these compounds to inhibit neurodegeneration in the hippocampus and cortex regions [136]. Moreover, proanthocyanidins, a class of natural flavonoids, proved to mitigate rotenone-induced oxidative stress in human neuroblastoma SH-SY5Y dopaminergic cells, a model for Parkinson’s disease [137]. Similarly, one study investigated the effects of standardized flavonoid extracts of safflower on a 6-hydroxydopamine-induced Parkinson’s disease rat model. The results showed that the administration of this extract could improve behavioral performances through the suppression of reactive astrogliosis and α-synuclein overexpression and aggregation [138]. The anti-neuroinflammatory effects of flavonoids have also been studied, proving the potential of tiliroside, a natural dietary glycosidic flavonoid, to increase the protein levels of Nrf2, HO-1 and NQO1, indicating an activation of the Nrf2 protective mechanisms in the microglia [139], and the potential of rutin and quercetin isolated from Glaucium corniculatum extracts to inhibit the secretion of IL-6 and IL-10 cytokines in H_2_O_2_-stimulated PC12 cells [140]. Flavonoids have also proved to play fundamental roles in the therapy of ischemic stroke. Specifically, the administration of 2′-methoxy-6-methylflavone increases GABA_A_ receptor tonic currents, which are elevated following stroke, through the δ-containing GABA_A_ receptors, decreases infarct volume, and improves functional recovery [141]. Additionally, the administration Ilex Pubescens total flavonoids on neuroprotection using a rat model of focal cerebral ischemia/reperfusion injury has led to a reduction of the neurological deficit score, infarcted area, and brain tissue pathological injury, an increase of the brain-derived neurotrophic factor, glial cell-derived neurotrophic factor and vascular endothelial growth factor expression levels, and a decrease of various proinflammatory cytokines [142].

The category of non-flavonoids comprises phenolic compounds with variated chemical structures, of which the most important are phenolic acids, that contain a single phenol unit substituted by one carboxylic group and at least one hydroxyl group [120]. Other non-flavonoids classes include tannins, coumarins, lignans, quinones, stilbens, and curcuminoids [117,121]. Phenolic acids are biosynthesized through the shikimate pathway and they consist of two main classes, namely hydroxybenzoic acids and hydroxycinnamic acids, derived from benzoic and cinnamic acid, respectively [117]. These compounds are chemically heterogenous with varying properties, exhibiting high antioxidant activities [122]. In the central nervous system, phenolic acids have proved to exhibit neuroprotective effects by ameliorating ischemia, neuroinflammation, glutamate-induced toxicity, apoptosis, depression, memory-impairment, and hearing and vision disturbances. Their potential role in protecting neurons and glial cells has gained a great scientific interest in the last years [143]. Dietary polyphenols have shown to reduce the oxidative stress involved in the onset and progression of neurodegeneration. In this context, one group investigated the effects of a panel of polyphenols, including 3,4-dihydroxyphenylpropionic acid, 3,4-dihydroxyphenylacetic acid, gallic acid, and ellagic acid, at physiologically relevant concentrations against H_2_O_2_-induced oxidative stress on human neuroblastoma SH-SY5Y cells. The administration of these compounds has prevented neuronal apoptosis by attenuating ROS levels, preventing caspase-3 activation, and increasing redox activity [144]. Similarly, helisterculins C and D, two new phenolic acids were isolated from the aqueous extract of Costus spicatus, along with four other compounds, danshensu, rosmarinic acid, ethyl rosmarinate, and lithospermic acid. The neuroprotective potential of helisterculins C and D against 6-hydroxydopamine-induced cell death in SH-SY5Y cells proved to be moderate, but exhibited lower IC50 values when compared to the standard drug curcumin [145]. Another study reported the neuroprotective effect of gallic acid isolated from Sanguisorbae radix extracts against amyloid β-protein induced toxicity in rat cortical neurons cultures [146]. Ellagic acid was also studied for its antioxidant and anti-inflammatory effects on 6-hydroxydopamine rat model of Parkinson’s disease. The results showed attenuated apomorphine-induced rotational bias, reduced striatal malondialdehyde, ROS, and DNA fragmentation, increased levels of MAO-B, Nrf2, and haem oxygenase 1, and a limited loss of tyrosine hydroxylase-positive neurons within substantia nigra pars compacta [147]. Furthermore, wine-derived phenolic compounds, namely 3,4-dihydroxyphenylacetic, 3-hydroxyphenylacetic acid, and salicylic β-d-O-glucuronide, have exhibited neuroprotective effects on SH-SY5Y neuroblastoma cells by inhibiting caspase-3 activity and prevention of RNS-induced stress injury [148]. The administration of carnosic acid, a phenolic compound isolated from Rosmarinus officinalis on a paraquat model of Parkinson’s disease, has led to decreased ROS/RNS levels, silenced Nrf2 expression, and reduced toxic effects on mitochondrial function [149]. Moreover, the efficiency of rosmarinic acid against memory deficits caused by permanent middle cerebral artery occlusion was tested on mice models. The results showed that the phenolic acid improved working, spatial, recognition memory deficits and reduced the infarct size and neuronal deficits through neuronal loss suppression and a synaptophysin expression increase [150].

### 4.3. Carotenoids

Carotenoids are a family of lipid-soluble pigmented compounds synthesized primarily in plants and algea, but also by microorganisms, such yeasts, fungi, archea, and eubacteria. Although they cannot be synthesized by animals, carotenoids are found in the animal kingdom through the selective absorption along the food chain [151,152,153]. In plants, carotenoids are de novo synthesized in the differentiated plastids of roots, flowers, seeds, and fruits, and their accumulation can be divided into chloroplasts, elaioplasts, leucoplasts, and etioplasts [154]. Carotenoids are fundamentally important in the photosynthesis process, protecting plants against light damage [152,153].

Carotenoids are characterized by a chemical structure comprising a long polyenic carbon chain of conjugated double carbon-carbon bonds which interact with each other and cause electrons to move freely across the molecule [153,155] and a nearly bilateral symmetry around the core [152]. The isoprenoid skeleton and its diverse foldings lead to the formation of numerous different structures with varied physical, chemical, and biological properties, along with diverse red, orange, and yellow colors [153]. Their chemical structure is highly responsible for their ability to interact with free radicals, offering them their antioxidant properties [156]. Therefore, besides their role in the photosynthetic organisms, carotenoids have shown to exhibit many health benefits in the human body when consumed in sufficient levels. Specifically, carotenoid intake could reduce the risk of developing various types of cancer and cardiovascular, eye, or other chronic diseases [152,153,155]. Additionally, some compounds exhibit provitamin A activity [157,158]. Almost 90% of the carotenoids in the human body are α- and β-carotene, lycopene, lutein, and cryptoxanthin, and their absorption depends on the discharge of nutrients from the food matrix, solubilization, and formation of micelles, as they are hydrophobic in nature [152,159].

Lycopene, a naturally occurring carotenoid with anti-inflammatory properties, has been studied for its potential to treat spinal cord ischemia/reperfusion injury in rats. The results showed the neuroprotective effects through the inhibition of neuroinflammation by suppressing cyclooxygenase-2, nuclear factor-κB, activate protein-1, and haem oxygenase-1 [160]. Additionally, its neuroprotective effects have been investigated in aluminum chloride-induced hippocampal lesions in rats. The administration of lycopene has led to the alleviation of cognition impairment and oxidative stress by reducing malondialdehyde and 8-hydroxy-2′-deoxyguanosine levels and increasing glutathione level and superoxide dismutase activity, which further prevented neuroinflammation and apoptosis [161]. Similarly, astaxanthin, a xanthophyll carotenoid compound, has proved to have neuroprotective properties by: Inhibiting lipopolysaccharide-induced neuroinflammation, oxidant activity, and amyloidogenesis in mice models [162]; preventing hippocampal insulin resistance and Alzheimer’s disease complications in Wister rats [163]; preventing brain damage in offspring exposed to prenatal maternal seizures due to epilepsy [164]. Moreover, the co-administration of astaxanthin and fucoxanthin exhibited neuroprotective effects on pheochromocytoma neuronal cells for the treatment of Alzheimer’s disease [165]. Another carotenoid with antioxidant properties, crocin, has been administered for the therapy of Alzheimer’s and Parkinson’s diseases with the results proving its potential in the treatment of neurodegeneration [166,167,168]. Furthermore, one studied investigated the potential of β-carotene for the treatment of acute spinal cord injury with the results showing the reduced progression of secondary injury events by preventing the nuclear factor–κB pathway [169].

## 5. Conclusions and Future Perspectives

As oxidative stress is the major factor involved in the onset of neurodegenerative disorders due to the highly increased metabolical activity and reduced cellular regeneration capacity of the brain, scientists are focusing on the development of therapeutic strategies that could counteract the action of ROS and RNS. While the human body possesses an innate antioxidant defense mechanism which aims to prevent the reaction between free radicals and biological compounds, the exogenous administration of antioxidative compounds is fundamental. In this manner, several dietary antioxidants have been investigated for their neuroprotective effects in the treatment of neurodegenerative disorders. The most common exogenous antioxidant compounds include vitamins, phenolic compounds, and carotenoids, which are abundantly found in plants. However, novel strategies should be developed in order to maximize their effect. Considering the instability of natural antioxidants, such strategies could involve the development of drug delivery systems employing nanovehicles for the controlled release of these molecules at the specific site. Moreover, the co-administration of antioxidants with different directions of treatment could represent a potential solution to the limited efficacy of current therapies. Additionally, recent studies have shown that the controlled modulation of reactive sulfur species, such as H_2_S, could exert beneficial and synergistic effects with ROS/RNS inhibitors, acting as neuroprotective agents [170,171,172].

## Figures and Tables

**Table 1:** The most common antioxidant molecules in neuroprotection and their chemical structure.

Class of Compounds		Compound Name		Chemical Structure
**Vitamins**		Vitamin A	retinol	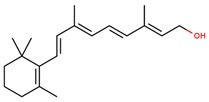
			retinal	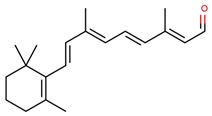
			retinoic acid	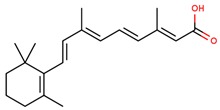
		Vitamin E (α-tocopherol)		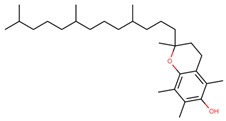
		Vitamin C (ascorbic acid)		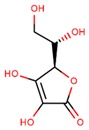
Phenolic compounds	Flavonoids	amurensin		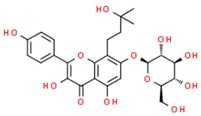
		cosmosiin		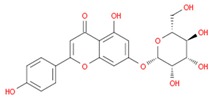
		tiliroside		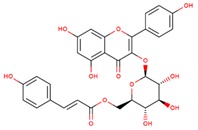
		rutin		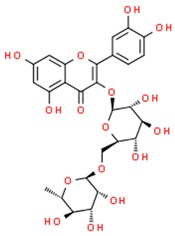
		quercetin		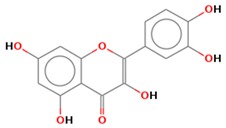
		2′-methoxy-6-methylflavone		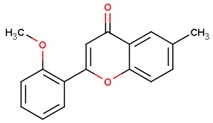
	Non-flavonoids	3,4-dihydroxyphenylpropionic acid		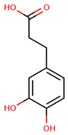
		3,4-dihydroxyphenylacetic acid		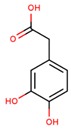
		gallic acid		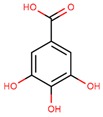
		ellagic acid		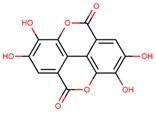
		3-hydroxyphenylacetic acid		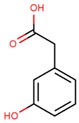
		salicylic β-d-O-glucuronide		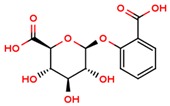
		carnosic acid		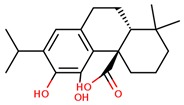
		rosmarinic acid		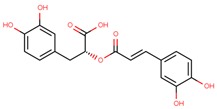
Carotenoids		α-carotene		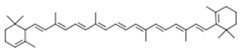
		β-carotene		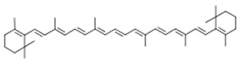
		lycopene		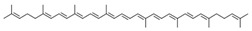
		lutein		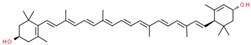
		cryptoxanthin